# Effects of physical activity on the development and progression of microvascular complications in type 1 diabetes: retrospective analysis of the DCCT study

**DOI:** 10.1186/1472-6823-13-37

**Published:** 2013-10-02

**Authors:** Caroline BT Makura, Krishnarajah Nirantharakumar, Alan J Girling, Ponnusamy Saravanan, Parth Narendran

**Affiliations:** 1 School of Clinical and Experimental Medicine, University of Birmingham, Birmingham, UK; 2 School of Health and Population Sciences, University of Birmingham, Birmingham, UK; 3 Division of Metabolic & Vascular Health, University of Warwick, Warwick, UK; 4 Department of Diabetes, University Hospital Birmingham, Birmingham, UK; 5 Room 229, Institute of Biomedical Research, The Medical School, University of Birmingham, Edgbaston, Birmingham B15 2TT, UK

**Keywords:** Physical activity, Exercise, Complications, Retinopathy, Type 1 diabetes

## Abstract

**Background:**

To examine the effects of physical activity on the development and progression of microvascular complications in patients with type 1 diabetes.

**Methods:**

A retrospective analysis of data from the Diabetes Control and Complications trial was undertaken. Physical activity data was collected at baseline for each of 1441 recruits, converted to metabolic equivalent of task values, and categorised according to the American College of Sports Medicine recommendations. The rates of development/progression of diabetic retinopathy, nephropathy and neuropathy were compared in those who achieved over twice recommended, up to twice recommended, and less than recommended metabolic equivalent of task levels of activity. The DCCT study had a mean duration of follow up of 6.5 years ending in 1993.

**Results:**

A total of 271 subjects had a sustained three-step progression in diabetic retinopathy. The rates of development or progression of retinopathy showed no significant association with physical activity level. The number of outcomes for nephropathy and neuropathy were small and there was no significant association with physical activity level.

**Conclusions:**

We found no evidence that physical activity improves microvascular outcomes in type 1 diabetes. However we demonstrate no evidence of harm. We suggest that physical activity continues to play an important role in the management of type 1 diabetes.

## Background

The evidence for benefit of physical activity in type 1 diabetes is poorly delineated. Whilst it improves wellbeing, lipid profile and macrovascular disease in type 1 diabetes [1,2], there is limited evidence for benefit to blood pressure, glucose control or microvascular diseases such as retinopathy, nephropathy and neuropathy [3]. We set out to determine whether physical activity protects against microvascular complications, the consequences of diabetes that are often patients’ biggest fear [4,5].

To date, cross-sectional studies have demonstrated that microvascular complications are associated with decreased physical activity in patients with type 1 diabetes [6]. Causality however has not been demonstrated, and this association could be explained by the presence of complications impairing the ability to undertake physical activity, rather than physical activity decreasing the complications of diabetes. In one study where adult patients with type 1 diabetes were asked to estimate their physical activity during teenage years have reported an inverse relationship between activity and the subsequent development of nephropathy and neuropathy [7]. However this inverse association was seen only in males, and was not seen for retinopathy. Although this study controlled for a number of important factors such as duration of diabetes, and baseline HbA1c, the subjective and retrospective estimation of physical activity weakens these findings.

To examine whether physical activity protects against microvascular complications in a more accurate and longitudinal fashion, we chose to examine publicly available high quality data from the Diabetes and Complications Trial (DCCT) [8]. This is a large randomised controlled trial originally designed to examine whether good glucose control prevents microvascular complications. The study also provides detailed physical activity levels as well as accurate data on all the other predictors of microvascular diseases in Type 1 diabetes. No other prospective dataset exists, and it is therefore a powerful tool with which to examine the area of interest.

## Methods

### Ethics statement

Ethical approval was obtained for each of the 29 study centres across USA and Canada for the DCCT study from the Hospital for Sick Children Research Ethics Board. All study participants provided written informed consent.

### Study design, participants and intervention

Anonymised data from the Diabetes Control and Complication Trial (DCCT) [8] was obtained with ethical approval. Here, patients with type 1 diabetes aged between 13 to 39 years were divided into either the primary (726pts) or secondary (715pts) prevention cohort. The primary prevention cohort had no evidence of microvascular complications, and diabetes for less than 5 years. Those in the secondary prevention cohort had diabetes less than 15 years, minimal-moderate retinopathy and urinary albumin excretion rate of less than 200 mg per 24 hours. Each cohort was randomised to receive either intensive or standard glucose lowering therapy.

### Exposure of interest (Leisure time physical activity)

At entry into the study, each patient was asked to specify the amount of time spent in leisure time physical activity (LTPA) of different intensities for the previous seven days. These activities were converted to Metabolic Equivalent of Task (MET) values - the median MET value for each level of activity described in the questionnaire was calculated using previously defined criteria [9] and used for data analysis. According to the international classification by Ainsworth used by American College of Sports Medicine (ACSM), 'light’ activity was allocated 3 METs, 'moderate’ activity (defined as 3–6 METs) was allocated of 4 METs, 'hard activity’ 6 METs and 'very hard activity’ 9 METs. For each participant, we multiplied this allocated median MET value by the time (minutes) spent in that activity to obtain the MET for that level of activity. The sum of METs from all activities was recorded as the total leisure time activity for each participant at entry into the DCCT. All participants were categorised into three groups based on the ACSM recommendation for METs.min/week (450-750METs) [10]: those who did not achieve the recommended level of METs for a given week (<450METs), those achieving recommended to twice the recommended level (450-1500METs), and those achieving more than twice the recommended METS (>1500METs).

### Outcome measure

Progression of retinopathy was defined to have occurred if there was a sustained (6 months) change of three steps or more from baseline on the Early Treatment Diabetic Retinopathy Study (ETDRS) scale [11]. Nephropathy was defined as albumin excretion rate (AER) of >40 mg/24 hour [12,13]. Each participant was examined quarterly every year and the time to progression of retinopathy and occurrence of nephropathy was recorded. Neuropathy was assessed by clinical examination and confirmed by either abnormal nerve conduction or autonomic nervous system testing or both at a fixed time point of 5 years follow up [14]. Patients with nephropathy and neuropathy at baseline were excluded from the analysis in order to study onset of complications [13,14].

Covariates included duration of diabetes, baseline HbA1c, triglycerides, cholesterol, diastolic and systolic blood pressure, BMI and smoking status. Occupational activity representing physical activity at work, school or home was also recorded by DCCT specific questionnaire as three categories namely sedentary (such as office work with occasional inter-office walking), moderate (work requiring considerable but not constant, lifting, walking, bending and pulling) and strenuous activity (requires almost constant lifting, bending, pulling, scrubbing etc.).

The DCCT study was followed by a subsequent study called Epidemiology of Diabetes Interventions and Complications (EDIC). Here the patients who had participated in the DCCT study were unblinded and asked to remain in follow-up to determine the effect of the DCCT intervention on complications in the longer term. We did not consider using the EDIC dataset because the exposure of interest was at DCCT baseline and therefore the time interval between exposure and outcome was too long to make a meaningful interpretation. Furthermore the EDIC data only recorded presence of outcome and did not have data on time to event for retinopathy or nephropathy.

### Statistical analysis

Analysis was carried out separately for each of the intensive and standard glucose lowering therapy arms of the DCCT trial. We chose to analyse the data in this way because glucose lowering is now established to protect from microvascular complications and would have been an important confounder. One-way analysis of variance test was used to compare continuous variables and the chi-square test was used to compare the categorical variables in the three LTPA groups.

Kaplan Meier survival curves (with the retinopathy and nephropathy as endpoint) were generated for the three LTPA groups stratified according to the primary and secondary prevention cohort. Initially we used log-rank test in each arm stratified according to the prevention cohort to compare LTPA groups. This was supplemented by a multivariate Cox regression analysis with stratification by primary and secondary prevention cohort. In this regression model we controlled for age, gender, duration of diabetes, baseline HbA1c value and occupational activity.

Given the smaller number of outcomes and fixed time point for neuropathy, only a univariate analysis (Chi-square test) was performed. Analysis was conducted in the SPSS 18 statistical package.

## Results and discussion

The baseline characteristics of the patients enrolled in the study are shown in Table 1 stratified according to trial arm. Mean age of all 1441 participants was 26.8 years with 52.8% being males. Interestingly, the population appears to be very active with only 20% of the patient group not meeting recommended levels of activity. Follow-up was for a mean of 6.5 years (range 3 to 9). LTPA was positively associated with younger age in both arms of the trial (P <0.01), and with male sex and non-smoking status in the intensive arm of the trial. There was no association between LTPA and HbA1c, cholesterol, triglycerides, blood pressure or BMI at trial entry (P>0.05). There was no difference in LTPA between the standard and intensive arms of the trial.

**Table 1:** Baseline characteristics of the leisure time physical activity groups in intensive and standard arms of DCCT study

	**Standard treatment**				**Intensive treatment**			
	**Category1 (N=130)**	**Category 2 (N=194)**	**Category 3 (N=387)**	**P value**	**Category 1 (N=141)**	**Category 2 (N=207)**	**Category 3 (N=382)**	**P value**
^**#**^**Age (Years)**	28.7(5.9)	27.8(6.8)	25.0(7.4)	<0.01	29.4(6.1)	28.2(6.5)	25.8(7.4)	<0.01
^**#**^**Duration of diabetes (Months)**	62.8(50.0)	67.4(49.2)	65.7(48.6)	0.70	74.7(51.3)	69.2(51.0)	68.2(50.4)	0.45
^**#**^**HbA1c**	9.1(1.9)	9.0(1.6)	9.1(1.5)	0.66	9.3(1.7)	8.9(1.5)	9.1(1.6)	0.18
^**#**^**Cholesterol(mg/dl)**	179.6(36.1)	177.5(34.8)	173.3(31.8)	0.11	176.3(30.3)	179.1(34.7)	176.4(32.7)	0.62
^**#**^**Triglycerides(mg/dl)**	82.4(54.0)	81.0(42.3)	82.0(54.8)	0.97	77.7(43.2)	78.5(38.1)	83.1(45.6)	0.32
^**#**^**BMI (Kg/m2)**	23.5(2.9)	23.5(2.9)	23.4(2.9)	0.88	23.3(2.8)	23.4(2.7)	23.3(2.7)	0.76
^**#**^**DBP (mmHg)**	72.6(8.6)	72.6(8.7)	73.0(9.0)	0.82	71.5(8.7)	72.5(8.9)	72.5(8.9)	0.51
^**#**^**SBP (mmHg)**	113.0(11.5)	114.7(11.3)	115.4(12.0)	0.12	113.1(11.3)	113.5(12.0)	113.4(11.4)	0.95
*Primary prevention cohort %	57.4	46.9	52.4	0.14	48.5	46.9	50.1	0.76
*Male %	47.5	54.1	56.5	0.19	40.8	43.8	58.9	<0.001
*Current smoker %	29.1	22.7	18.3	0.11	26.9	20.6	18.3	0.03
*Occupational activity								
**Sedentary %**	37.6	33.3	26.4	0.06	29.2	35.6	26.7	0.09
**Moderate %**	53.9	59.9	66.8		64.6	58.8	64.8	
**Vigorous %**	8.5	6.8	6.8		6.2	5.7	8.5	

Category 1 = METS less than 450Category 2 = METS 450-1500Category 3 = METS > 1500. # Mean (Standard Deviation).

* Percentage.

**Figure 1:**
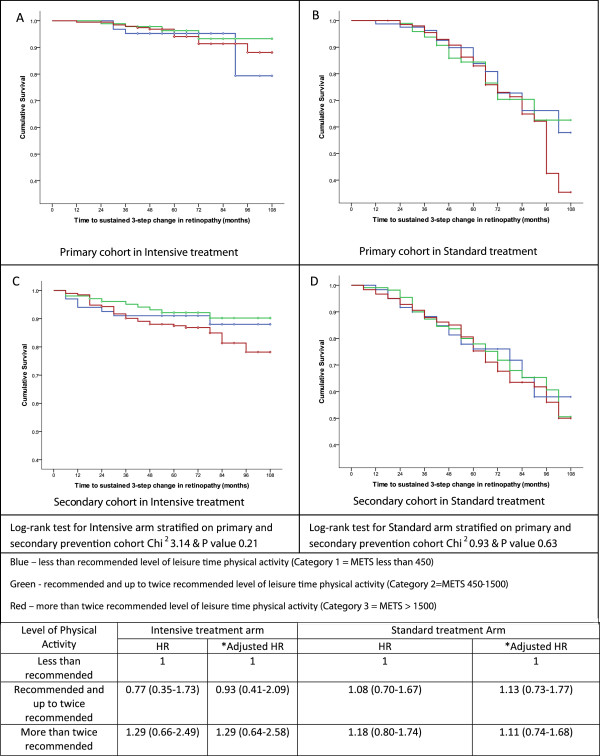
**Kaplan-Meier survival curves and hazard ratios for retinopathy in the DCCT dataset according to LPA (data is categorised according to Standard / Intensive treatment arm, and Primary / Secondary prevention cohort).** Data is categorised according to Standard / Intensive treatment arm, and Primary / Secondary prevention cohort and each curve is stratified according to the three categories of Metabolic Equivalent of Tasks. Panel **A** shows the primary cohort in the Intensive arm, Panel **B** shows the primary cohort in the standard treatment arm, Panel **C** shows the secondary cohort in the intensive treatment arm and Panel **D** shows the secondary cohort in the standard treatment arm. Both the survival curves in panels **A** and **C** show a higher cumulative survival rate when compared to the survival graphs in Panel **B** and **D**. However, Kaplan-Meier analysis of the survival graphs did not demonstrate any difference in progression of retinopathy in the three incremental physical activity categories in either the intensive or standard treatment arm. Log-rank test for the intensive arm Chi2 is 3.14 (P=0.21) and Log-rank test for the standard treatment arm Chi2 is 0.93 (P=0.63). Separation in the curves seen towards the end of the graphs is indicative of the fewer numbers followed up for a longer period.

Kaplan-Meir survival analysis did not reveal any difference in progression of retinopathy in the three incremental physical activity categories either in the intensive or standard treatment arm when stratified according to cohort (Log-rank test Intensive Chi2 3.14;P=0.21 and standard Chi2 0.93;P=0.63) (Figure 1). In the multivariate analysis the hazard ratios (HR) were not significant, either in the recommended to twice recommended (adjusted HR, 95%CI:0.93 (0.41-2.09)) or in the above twice recommended (adjusted HR, 95%CI:1.13 (0.73-1.77)) categories in the intervention arm. Similarly it was not significant in the standard treatment arm (Figure 1).

The numbers of subjects developing nephropathy and neuropathy were small (97 for nephropathy and 28 for neuropathy). We did however undertake an analysis of the effect of LTPA on the progression of these complications, but were not able to detect any association (see Additional files 1 and 2). Further analysis with all patients (combined primary/secondary cohort, standard/intensive treatments) provided similar results.

## Conclusion

In this large prospective interventional study, we observed the physical activity levels were higher than the average population [15]. Only 20% of the patients did not meet the recommended daily physical activity levels. This may be due to positive selection bias (patients volunteered are also motivated to do physical activity).

Current modifiable risk factors for microvascular complications in type 1 diabetes are recognised to be glycaemic control, blood pressure, lipids, insulin resistance and smoking [16-18]. Of these, glycaemic control is arguably the most powerful predictor of retinopathy. There is no conclusive evidence for the role of physical activity on microvascular complications. In this detailed analysis of the DCCT study, we also found no evidence that leisure time physical activity protects against either the development or progression of microvascular complications in type 1 diabetes. This lack of benefit in our analysis may be explained by the weak glycaemic benefit of exercise in type 1 diabetes [19-21]. We also speculate such lack of benefit may also be due to temporary deterioration in the glycaemic control due to reduction in insulin doses to avoid hypoglycaemia, though no such data is available in the study. However, we believe this study is better suited to examining the effect of exercise on the progression of microvascular complications, than previous cross sectional [6] and retrospective studies [7], whose results it contradicts.

The main limitation of our study is the fact that it is a post hoc analysis. The DCCT study is neither designed nor powered to test whether physical activity protects from microvascular complications. Despite the lack of validation of the physical activity types at data collection, the study is unique as the leisure time and occupational activity data in the study are detailed. Another limitation is that the levels of physical activity may have varied during the course of the study, making a baseline assessment unrepresentative. Whilst the DCCT is now a dated study and diabetes practices will likely have changed in the past 20 years, we do not believe this invalidates the use of the dataset to examine our research question.

In summary, our study does not support a benefit of physical activity on microvascular complications in patients with type 1 diabetes. However, a prospective randomised controlled study will be required to address this question adequately. In the mean time, it would be advisable to continue to support a program of physical activity in patients with type 1 diabetes for its benefits on wellbeing, macrovascular disease and mortality.

## Competing interests

All authors declared they have no competing interests associated with this manuscript.

## Authors’ contributions

CM: analysed the data, helped author the paper. KN: analysed the data, helped author the paper. AG: supported statistical analysis. PS: conceived the research idea and supported the writing. PN: conceived the research idea, obtained the data, and supported the data analysis and writing. All authors read and approved the final manuscript.

## Pre-publication history

The pre-publication history for this paper can be accessed here:

http://www.biomedcentral.com/1472-6823/13/37/prepub

## Supplementary Material

Additional file 1 **Kaplan-Meier survival curves and hazard ratios for nephropathy.** N=1365, sustained albuminuria >=40mg /24 hrs and excludes patients with albuminuria >=40mg/24 hrs at baseline in the DCCT dataset. Data is categorised according to Standard / Intensive treatment arm, and Primary / Secondary prevention cohort and each curve is stratified according to the three categories of Metabolic Equivalent of Tasks. Panel **A** shows the primary cohort in the Intensive arm, Panel **B** shows the primary cohort in the standard treatment arm, Panel **C** shows the secondary cohort in the intensive treatment arm and Panel **D** shows the secondary cohort in the standard treatment arm. Both the survival curves in panels **A** and **C** show a higher cumulative survival rate when compared to the survival graphs in Panel **B** and **D** with those in the primary cohort and Intensive arm demonstrating the highest cumulative survival rate. A total of 97 subjects developed nephropathy. Kaplan-Meier analysis of the survival graphs suggested a difference in progression of nephropathy for the Standard arm stratified on primary and secondary prevention cohort in the three incremental physical activity categories (Log rank test: Chi2 6.61, P=0.04). However in the adjusted multivariate cox regression analysis there was no demonstrable significant associations (Hazard ratio 0.52 (0.22-1.21) for recommended and up to twice recommended and 0.99 (0.49-1.98) for more than twice recommended). There were no significant trend or associations noted in the more relevant intensive treatment arm. Click here for file

Additional file 2 **Neuropathy analysis based on 5 year outcome of neuropathy as defined by DCCT group.** 1161 patients, who were free of neuropathy at baseline were followed. A total of 108 patients presented with neuropathy at 5 years of which 28 of were in the Experimental treatment arm and 80 in the Standard treatment arm. Analysis was carried out in the Control arm and Treatment arm tables stratified on primary and secondary prevention cohort in the three incremental physical activity categories. There were no demonstrable association noted in either arm of the study (p>0.05). Click here for file
