# Supplementary material for: Effects of physical activity on the development and progression of microvascular complications in type 1 diabetes: retrospective analysis of the DCCT study
Source: BMC Endocr Disord. 2013 Oct 2;13:37. doi: 10.1186/1472-6823-13-37 (PMC3850661; doi:10.1186/1472-6823-13-37)
Supplement: Additional file 1 — Kaplan-Meier survival curves and hazard ratios for nephropathy. N=1365, sustained albuminuria >=40mg /24 hrs and excludes patients with albuminuria >=40mg/24 hrs at baseline in the DCCT dataset. Data is categorised according to Standard / Intensive treatment arm, and Primary / Secondary prevention cohort and each curve is stratified according to the three categories of Metabolic Equivalent of Tasks. Panel A shows the primary cohort in the Intensive arm, Panel B shows the primary cohort in the standard treatment arm, Panel C shows the secondary cohort in the intensive treatment arm and Panel D shows the secondary cohort in the standard treatment arm. Both the survival curves in panels A and C show a higher cumulative survival rate when compared to the survival graphs in Panel B and D with those in the primary cohort and Intensive arm demonstrating the highest cumulative survival rate. A total of 97 subjects developed nephropathy. Kaplan-Meier analysis of the survival graphs suggested a difference in progression of nephropathy for the Standard arm stratified on primary and secondary prevention cohort in the three incremental physical activity categories (Log rank test: Chi2 6.61, P=0.04). However in the adjusted multivariate cox regression analysis there was no demonstrable significant associations (Hazard ratio 0.52 (0.22-1.21) for recommended and up to twice recommended and 0.99 (0.49-1.98) for more than twice recommended). There were no significant trend or associations noted in the more relevant intensive treatment arm. [file 1472-6823-13-37-S1.pdf]

Appendix 1: Kaplan-Meier survival curves and hazard ratios for nephropathy (N=1365, sustained albuminuria >=40mg /24 hrs) in the DCCT dataset according to LPA (data is categorised according to Standard / Intensive treatment arm, and Primary / Secondary prevention cohort). Excludes patients with albuminuria >=40mg/24 hrs at baseline

A

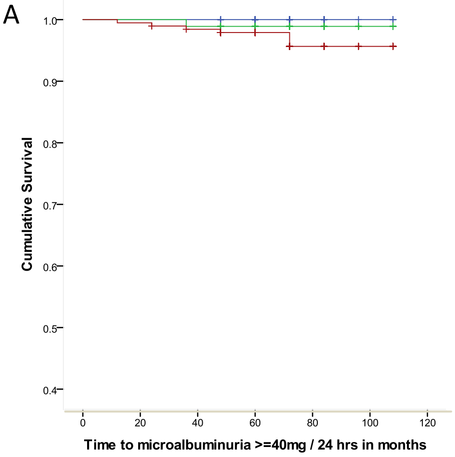

Primary cohort in Intensive treatment

B

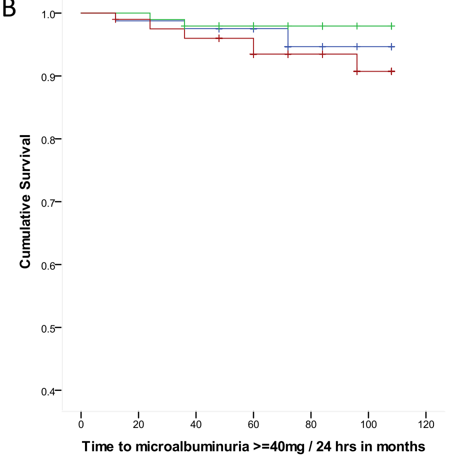

Primary cohort in Standard treatment

C

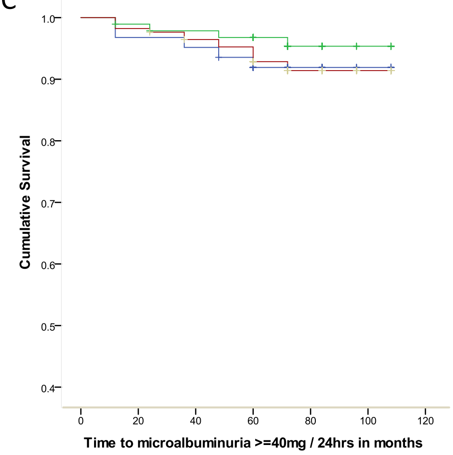

Secondary cohort in Intensive treatment

D

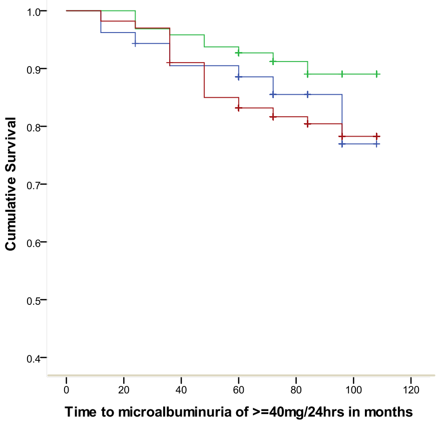

Secondary cohort in Standard treatment

Log-rank test for **Intensive arm** stratified on primary and secondary prevention cohort **Chi^2 2.61 & P value 0.27**

Log-rank test for **Standard arm** stratified on primary and secondary prevention cohort **Chi^2 6.61 & P value 0.04**

Blue – less than recommended level of leisure time physical activity (**Category 1 = METS less than 450)**

Green - recommended and up to twice recommended level of leisure time physical activity **(Category 2=METS 450-1500)**

Red – more than twice recommended level of leisure time physical activity **(Category 3 = METS > 1500)**

Level of Physical Activity	Intensive treatment arm		Standard treatment Arm	
	HR	*Adjusted HR	HR	*Adjusted HR
Less than recommended	1	1	1	1
Recommended and up to twice recommended	0.66 (0.19-2.27)	0.75 (0.21-2.62)	0.59 (0.26-1.36)	0.52 (0.22-1.21)
More than twice recommended	1.41 (0.53-3.75)	0.98 (0.34-2.84)	1.34 (0.70-2.60)	0.99 (0.49-1.98)

*Adjusted for age, gender, duration of diabetes, HbA1c and occupational activity
