# Supplementary material for: Effects of physical activity on the development and progression of microvascular complications in type 1 diabetes: retrospective analysis of the DCCT study
Source: BMC Endocr Disord. 2013 Oct 2;13:37. doi: 10.1186/1472-6823-13-37 (PMC3850661; doi:10.1186/1472-6823-13-37)
Supplement: Additional file 2 — Neuropathy analysis based on 5 year outcome of neuropathy as defined by DCCT group. 1161 patients, who were free of neuropathy at baseline were followed. A total of 108 patients presented with neuropathy at 5 years of which 28 of were in the Experimental treatment arm and 80 in the Standard treatment arm. Analysis was carried out in the Control arm and Treatment arm tables stratified on primary and secondary prevention cohort in the three incremental physical activity categories. There were no demonstrable association noted in either arm of the study (p>0.05). [file 1472-6823-13-37-S2.pdf]

Appendix 2: Neuropathy: Analysis based on 5 year outcome of neuropathy as defined by DCCT group*

Patients with neuropathy at baseline were excluded from the analysis

Experimental arm

Primary and Secondary cohort			Neuropathy at 5 year follow-up: No	Neuropathy at 5 year follow-up: Yes	Total
Primary		Less than recommended LPA	46<br>95.8%	2<br>4.2%	48<br>100.0%
		Recommended and twice recommended	62<br>96.9%	2<br>3.1%	64<br>100.0%
		Above twice recommended	133<br>97.8%	3<br>2.2%	136<br>100.0%
	Total		241<br>97.2%	7<br>2.8%	248<br>100.0%
Secondary		Less than recommended LPA	52<br>89.7%	6<br>10.3%	58<br>100.0%
		Recommended and twice recommended	81<br>92.0%	7<br>8.0%	88<br>100.0%
		Above twice recommended	161<br>95.3%	8<br>4.7%	169<br>100.0%
	Total		294<br>93.3%	21<br>6.7%	315<br>100.0%
Total		Less than recommended LPA	98<br>92.5%	8<br>7.5%	106<br>100.0%
		Recommended and twice recommended	143<br>94.1%	9<br>5.9%	152<br>100.0%
		Above twice recommended	294<br>96.4%	11<br>3.6%	305<br>100.0%
	Total		535<br>95.0%	28<br>5.0%	563<br>100.0%

Chi-Square Tests

Primary and Secondary		Value	df	Asymp. Sig. (2-sided)
Primary	Pearson Chi-Square	.526[a]	2	.769
Secondary	Pearson Chi-Square	2.510[b]	2	.285
Total	Pearson Chi-Square	2.980[c]	2	.225

Control Arm

Primary and Secondary cohort			Neuropathy at 5 years No	Neuropathy at 5 years Yes	Total
Primary		Less than recommended LPA	52	10	62
			83.9%	16.1%	100.0%
		Recommended and twice recommended	63	7	70
			90.0%	10.0%	100.0%
		Above twice recommended	148	11	159
			93.1%	6.9%	100.0%
	Total		263	28	291
			90.4%	9.6%	100.0%
Secondary		Less than recommended LPA	45	7	52
			86.5%	13.5%	100.0%
		Recommended and twice recommended	78	19	97
			80.4%	19.6%	100.0%
		Above twice recommended	132	26	158
			83.5%	16.5%	100.0%
	Total		255	52	307
			83.1%	16.9%	100.0%
Total		Less than recommended LPA	97	17	114
			85.1%	14.9%	100.0%
		Recommended and twice recommended	141	26	167
			84.4%	15.6%	100.0%
		Above twice recommended	280	37	317
			88.3%	11.7%	100.0%
	Total		518	80	598
			86.6%	13.4%	100.0%

Chi-Square Tests

Primary and Secondary		Value	df	Asymp. Sig. (2-sided)
Primary	Pearson Chi-Square	4.367[a]	2	.113
Secondary	Pearson Chi-Square	.957[b]	2	.620
Total	Pearson Chi-Square	1.720[c]	2	.423
